# Twelve years of SAMtools and BCFtools

**DOI:** 10.1093/gigascience/giab008

**Published:** 2021-02-16

**Authors:** Petr Danecek, James K Bonfield, Jennifer Liddle, John Marshall, Valeriu Ohan, Martin O Pollard, Andrew Whitwham, Thomas Keane, Shane A McCarthy, Robert M Davies, Heng Li

**Affiliations:** Wellcome Sanger Institute, Wellcome Genome Campus, Hinxton, Cambridgeshire CB10 1SA, UK; Wellcome Sanger Institute, Wellcome Genome Campus, Hinxton, Cambridgeshire CB10 1SA, UK; Wellcome Sanger Institute, Wellcome Genome Campus, Hinxton, Cambridgeshire CB10 1SA, UK; Wolfson Wohl Cancer Research Centre, Institute of Cancer Sciences, University of Glasgow, Switchback Road, Glasgow, G61 1QH, UK; Wellcome Sanger Institute, Wellcome Genome Campus, Hinxton, Cambridgeshire CB10 1SA, UK; Wellcome Sanger Institute, Wellcome Genome Campus, Hinxton, Cambridgeshire CB10 1SA, UK; Wellcome Sanger Institute, Wellcome Genome Campus, Hinxton, Cambridgeshire CB10 1SA, UK; EMBL-EBI, Wellcome Genome Campus, Hinxton, Cambridgeshire, CB10 1SD, UK; Wellcome Sanger Institute, Wellcome Genome Campus, Hinxton, Cambridgeshire CB10 1SA, UK; Wellcome Sanger Institute, Wellcome Genome Campus, Hinxton, Cambridgeshire CB10 1SA, UK; Department of Data Sciences, Dana-Farber Cancer Institute, 450 Brookline Avenue, Boston, MA 02215, USA; Department of Biomedical Informatics, Harvard Medical School, 10 Shattuck Street, Boston, MA 02215, USA

**Keywords:** samtools, bcftools, high-throughput sequencing, next generation sequencing, variant calling, data analysis

## Abstract

**Background:**

SAMtools and BCFtools are widely used programs for processing and analysing high-throughput sequencing data. They include tools for file format conversion and manipulation, sorting, querying, statistics, variant calling, and effect analysis amongst other methods.

**Findings:**

The first version appeared online 12 years ago and has been maintained and further developed ever since, with many new features and improvements added over the years. The SAMtools and BCFtools packages represent a unique collection of tools that have been used in numerous other software projects and countless genomic pipelines.

**Conclusion:**

Both SAMtools and BCFtools are freely available on GitHub under the permissive MIT licence, free for both non-commercial and commercial use. Both packages have been installed >1 million times via Bioconda. The source code and documentation are available from https://www.htslib.org.

## Background

With the advancement of genome sequencing technologies and large-scale sequencing projects, new data formats became necessary for interoperability, compact storage, and efficient analysis of the data. Among the most common formats used in this field today are SAM [1] and VCF [2], developed by the 1000 Genomes Project [3]. These specialized formats for storing read alignments (SAM) and genetic variants (VCF) are row-oriented tab-delimited text files, which are easy to process using custom scripts but slow to parse and can be inefficient to store. Therefore in practice, the binary counterparts BAM or CRAM are used for alignment data and, when parsing of large VCF files becomes prohibitively slow, BCF provides a more efficient format for processing variation data.

Despite the conceptual simplicity of the underlying DNA sequence, the alignment and variant data carry rich information. These data undergo a number of processing steps, many of which are algorithmically complex and require specialized software. Also more programming effort and expertise are necessary to parse binary formats. Therefore programs and toolkits that encompass functionality for the most common tasks have been developed. These include tools for file manipulation, quality control, and data analyses, such as sambamba [4], biobambam [5], FastQC [6], and GATK [7]. A successful bioinformatics tool must keep up with advancements in sequencing technologies (e.g., substantial increase in sequencing read length), scale well with ever-increasing amounts of data (from single to hundreds of thousands of genomes), and expand focus to encompass new analyses and more complex types of variation. New species also bring challenges such as large chromosomes not representable by 32 bits (>2 Gb) or assumptions about the ploidy of an organism. In this article we describe the status, new features, and developments in SAMtools and BCFtools.

SAMtools was originally published in 2009 [1]. Readers of the online edition of that article would have been able to download release 0.1.4. The package included not only utilities to convert and manipulate SAM and BAM files but also a variant caller, which was soon restructured into the BCFtools subpackage (2010, release 0.1.9). Later it became apparent that third-party projects were trying to use code from SAMtools despite it not being designed to be embedded in that way. Therefore the decision was taken in August 2014 (release 1.0) to split the SAMtools package into a stand-alone library with a well-defined API (HTSlib, currently 82k lines of code) [8], a project for variant calling and manipulation of variant data (BCFtools, 71k lines), and SAMtools for working with alignment data (42k lines). All 3 projects are maintained in parallel, and improvements to HTSlib naturally filter into new releases of SAMtools and BCFtools. Since the original release the combined size of HTSlib, SAMtools, and BCFtools has doubled.

## Findings

### SAMtools

Since the initial release there have been >2,200 commits to the code repository and 52 releases, the most recent being version 1.11 in September 2020 [9].

The main part of the SAMtools package is a single executable that offers various commands for working on alignment data. The “view" command performs format conversion, file filtering, and extraction of sequence ranges. Files can be reordered, joined, and split in various ways using the commands sort, collate, merge, cat, and split. Files can be indexed for fast random access using “index" for alignment files and “faidx" for reference sequences in the FASTA format. File content can be manipulated with commands like addreplacerg, calmd, fixmate, and reheader. Duplicated reads, caused by artefacts in the library creation and sequencing process, can be flagged using markdup. Various statistics on alignment files can be calculated using idxstats, flagstat, stats, depth, and bedcov. Data can be converted to legacy formats using fasta and fastq. For position-ordered files, the sequence alignment can be viewed using tview or output via mpileup in a way that can be used for ongoing processing (e.g., variant calling). Most recently SAMtools has gained support for amplicon-based sequencing projects via ampliconclip and ampliconstats.

For a complete list of SAMtools commands with a short summary, version, and date of the initial commit see Supplementary Table S1. Full documentation for these commands is included with the package in the form of UNIX man pages and can also be found online [10].

Early releases of SAMtools could read and write alignment data in the SAM and BAM formats. The 1.0 release introduced support for the better-compressed CRAM format [11]. Originally, the program required the use of command line options to select the input format, and most commands were tied to using BAM files. These restrictions were removed as SAMtools transitioned to use HTSlib, so by release 1.0 most commands could automatically detect the input file format and could directly read and write SAM, BAM, and CRAM files. In particular, there is rarely any need to convert SAM to BAM using “samtools view -b” before running commands like “samtools sort,” although regrettably this idiom still appears in a large number of online tutorials. We encourage readers to follow best practices and workflows published at [12].

SAMtools has also become faster, most notably by gaining the ability to use threads to take better advantage of the parallelism available on modern multicore systems. Thread support first arrived in version 0.1.19 (March 2013), which enabled them for sorting and BAM file writing in the view command. The number of tasks using threads has slowly increased, so now (thanks to improvements in HTSlib) it is possible to use them for both reading and writing SAM, BAM, and CRAM formats in most of the commands. Another time-saving improvement is the ability to index files as they are written (added in 1.10). This allows pipelines that need to index files to remove the separate “samtools index” stage and associated read-through of the file being indexed.

### BCFtools

The original purpose of the BCFtools package was to divide the I/O- and CPU-intensive tasks of variant calling into separate steps.

The first step, initially “samtools mpileup” but subsequently moved to “bcftools mpileup,” reads the alignments and for each position of the genome constructs a vertical slice across all reads covering the position (“pileup”). Genotype likelihoods are then calculated, representing how consistent are the observed data with the possible diploid genotypes. The calculation takes into account mapping qualities of the reads, base qualities, and the probability of local misalignment, per-base alignment quality (BAQ) [13]. The second step, “bcftools call” (known in the initial release as “bcftools view”), then evaluates the most likely genotype under the assumption of Hardy-Weinberg equilibrium (in the sample context customizable by the user) using allele frequencies estimated from the data or provided explicitly by the user. In 2016 (release 1.4) genotype likelihood generation was moved from SAMtools to BCFtools to make both variant-calling steps part of the same package and to prevent errors arising from the possible use of incompatible versions of the 2 programs.

Today BCFtools is a full-featured program that consists of 21 commands and 38 plugins (single-purpose tools) with >230 documented command line switches and options. As of writing, there have been >2,300 commits and 29 releases since 2012, with the most recent, 1.11, released in September 2020 [14].

The “bcftools view” command provides conversion between the text VCF and the binary BCF format, where both formats can be either plain (uncompressed) or block-compressed with BGZF for random access and compact size. The plain text VCF output is useful for visual inspection, for processing with custom scripts, and as a data exchange format. It should not be used when performance is critical because BCFtools internally uses the binary BCF representation and the conversion between the text VCF format and the binary BCF format is costly. Also compression and decompression is CPU intensive, and therefore when streaming between multiple commands in a pipeline it is recommended to stream uncompressed BCF by appending the option “-Ou.”

The program can do much more than convert between VCF and BCF formats. It can also process third-party formats (using the “convert" command) and manipulate variant files in many ways. It can be used to index, sort, and normalize variants (“norm"), replace headers (“reheader"), add and remove annotations (“annotate"), and subset samples (“view"). Most commands can filter sites either by a region, list of sites, or a general Boolean expression involving any combination of VCF tags (--include, --exclude). Multiple files can be compared, splitting common and file-specific variants into separate files according to custom rules (isec). Files sorted by position can also be combined using the merge command (input files have different samples) or concat command (input files have the same samples). Arbitrary fields can be extracted and formatted into a custom text output (query), a feature that, among other things, is useful for scripting.

Apart from file manipulation, the program offers variant callers and algorithms useful for analysis. For calling single-nucleotide polymorphisms and short indels from read alignment files, BCFtools implements 2 variant-calling models. In addition to the original biallelic caller (“bcftools call -c” [15]) there is a newer model available, capable of handling positions with multiple alternate alleles (“bcftools call -m”) and supporting gVCF output [16]. (For a recent comparison of the variant-calling component of the software see [17–20].) The package implements a hidden Markov model caller for detection of runs of homozygosity (roh [21]), copy-number variation calling from single-nucleotide polymorphism array data (cnv [22]), and the detection of whole-chromosome aberrations (polysomy). The program can construct a consensus sequence given a FASTA and a variant file (consensus), perform sample identity checks (gtcheck), and collect various statistics (stats).

In addition to built-in commands, the program supports a dynamic plugin mechanism for specific single-purpose tasks with a diverse range of functions. Examples from a large and ever-growing collection include the plugin split-vep for convenient querying and extraction of VEP annotations [23]; trio-dnm for ascertainment of *de novo* variants and their parental origin (parental-origin), or for collection of statistics (trio-stats) in trio data; gVCF manipulation (gvcfz); and many more.

For a complete list of BCFtools commands and plugins with a short summary, version, and date of the initial commit see Supplementary Table S2. Full documentation for these commands is included with the package in the form of UNIX man pages and can also be found online together with short tutorials, math notes, and other documentation at [24].

## Discussion

SAMtools and BCFtools represent a unique collection of tools useful for processing and analysis of sequencing data. Their development has been driven by the need of both large projects and individual user requests issued via GitHub. The code has been installed >1 million times via Bioconda [25] and GitHub releases [9, 14], and >1600 support and feature requests have been resolved on GitHub.

The programs are written in the C programming language and optimized for low memory consumption and high speed. For example, the “bcftools csq” command for prediction of functional consequences in a haplotype-aware manner requires only a fraction of the memory required by VEP and is 2 orders of magnitude faster [26].

Much work has been done to increase the reliability of SAMtools and BCFtools. The test harnesses now include ∼700 tests in SAMtools and ∼1,400 in BCFtools. Continuous integration services run all of the tests on a variety of platforms (including Linux, MacOS, and Windows) whenever code is checked into the source repository, ensuring that bugs are discovered and fixed rapidly. Code quality is also assured by checking for memory errors, originally using Valgrind memcheck [27] and more recently with AddressSanitizer [28]. Additionally, UndefinedBehaviorSanitizer is used to detect violations of the C standard.

Despite the ever-growing sample sizes and rapid increases in the amount of sequenced data, the programs have withstood the test of time. However, extremely big files are produced by large projects and their processing requires a high degree of parallelization on computing clusters. Future versions of SAMtools and BCFtools are expected to make more use of threaded code to allow faster processing of such files. Sometimes even the limits of BCF representation itself can be reached. For example, highly polymorphic sites can contain dozens of alternate indel alleles, which, in files with tens of thousands of samples, exceed the internal limit of 4 GB per site due to quadratic scaling of annotations such as FORMAT/PL. An extension of the VCF specification has been proposed to address this problem by introducing a localized version of such annotations with linear scaling [29] and has been implemented in BCFtools.

The programs have been used to process and analyze sequencing data from all types of species: vertebrates, invertebrates, pathogens, plants, and viruses. This provides interesting challenges and opportunities for future development. For example, some of the BCFtools commands are limited to handling haploid and diploid organisms and the support for large “64-bit” genomes is currently only partial. More work is also planned to overcome difficulties stemming from ambiguities in VCF allele encoding (such as operations of atomization and deatomization) and to improve visualization of results, and there are >50 feature requests currently registered on GitHub to investigate.

## Availability of Supporting Source Code and Requirements

Project name: SAMtools

Project home page: https://www.htslib.org, https://github.com/samtools/samtools

Operating system(s): Platform independent

Programming language: C

License: MIT/Expat

RRID:SCR_002105

biotools: samtools

Project name: BCFtools

Project home page: https://www.htslib.org, https://github.com/samtools/bcftools

Operating system(s): Platform independent

Programming language: C

Other requirements: Optional use of GNU Scientific Library (GSL)

License: MIT/Expat

RRID:SCR_002105

biotools: bcftools

## Data Availability

Snapshots of the code and tabular files are available from the *GigaScience* GigaDB repository [30].

## Editors Note

An accompanying article describing the HTSlib software library for providing programmatic access is published alongside this article [8].

## Additional Files

Supplementary Table S1: Table of SAMtools commands

Supplementary Table S2: Table of BCFtools commands

## Abbreviations

API: Application Programming Interface; CPU: central processing unit; GATK: Genome Analysis Toolkit; Gb: gigabase pairs.

## Competing Interests

The authors declare that they have no competing interests.

## Funding

This work was supported by a Wellcome Trust grant (206194).

## Authors' Contributions

J.K.B., P.D., R.M.D., H.L., J.M., M.O.P., V.O., and A.W. wrote the SAMtools software with J.L. and S.A.M. supporting; R.M.D., T.K., and J.M. provided supervision.

P.D. and S.A.M. wrote the BCFtools software with J.K.B., R.M.D., H.L., J.M., and V.O. supporting; S.A.M. also provided supervision.

J.K.B., P.D., R.M.D., V.O., and A.W. wrote the original draft of the manuscript with all authors reviewing.

## Supplementary Material

giab008_GIGA-D-20-00369_Original_Submission

giab008_GIGA-D-20-00369_Revision_1

giab008_Response_to_Reviewer_Comments_Original_Submission

giab008_Reviewer_1_Report_Original_Submission Fan Zhang -- 1/3/2021 Reviewed

giab008_Reviewer_2_Report_Original_Submission Chris Chang -- 1/6/2021 Reviewed

giab008_Supplemental_Files
