# Supplementary material for: Twelve years of SAMtools and BCFtools
Source: Gigascience. 2021 Feb 16;10(2):giab008. doi: 10.1093/gigascience/giab008 (PMC7931819; doi:10.1093/gigascience/giab008)
Supplement: giab008_Supplemental_Files [file giab008_supplemental_files.docx]

**Additional material for**

**Twelve years of SAMtools and BCFtools**

**Table S1.** **Table of SAMtools commands**

List of SAMtools commands with the date of the initial commit, and the version number of the release where the command became available. The coverage command was contributed by Florian Breitwieser, all others are the work of the authors. Some commands have been renamed - where this has happened, the first release that accepted the new name is noted. While the old names are no longer documented, in all cases they will be accepted as an alias for the new name if used.

| **Date** | **Version** | **Command** | **Description** |
| --- | --- | --- | --- |
| 2008-12-22 | 0.1.1 | faidx | index/extract FASTA |
| 2008-12-22 | 0.1.1 | merge | merge sorted alignments |
| 2008-12-22 | 0.1.1 | sort | sort alignment file |
| 2008-12-22 | 0.1.1 | tview | text alignment viewer |
| 2008-12-22 | 0.1.1 | view | SAM<->BAM<->CRAM conversion |
| 2008-12-22 | 0.1.1 | index | index alignment |
| 2009-01-22 | 0.1.2 | fixmate | fix mate information |
| 2009-01-29 | 0.1.3 | flagstat | simple stats |
| 2009-04-24 | 0.1.4 (as fillmd)  0.1.6 (as calmd) | calmd | recalculate MD/NM tags and '=' bases |
| 2010-06-11 | 0.1.8 | reheader | replace BAM header |
| 2010-06-12 | 0.1.8 | mpileup | multi-way pileup |
| 2010-06-13 | 0.1.8 | idxstats | BAM index stats |
| 2011-02-25 | 0.1.13 | phase | phase heterozygotes |
| 2011-02-25 | 0.1.13 | targetcut | cut fosmid regions (for fosmid pool only) |
| 2011-03-18 | 0.1.14 | cat | concatenate BAMs |
| 2011-04-01 | 0.1.15 | depth | compute the depth |
| 2012-02-08 | 0.1.19 | depad | convert padded BAM to unpadded BAM |
| 2012-04-17 | 0.1.19 | bedcov | read depth per BED region |
| 2012-05-17 | 0.1.19 (as bamshuf)  1.3 (as collate) | collate | shuffle and group alignments by name |
| 2013-09-09 | 1 | stats | generate stats (former bamcheck) |
| 2013-11-28 | 1 | flags | explain BAM flags |
| 2014-03-10 | 1 | split | splits a file by read group |
| 2014-04-17 | 1.0 (as bam2fq)  1.3 (as fastq) | fastq | converts a BAM to a FASTQ |
| 2015-03-19 | 1.3 | dict | create a sequence dictionary file |
| 2015-03-30 | 1.3 | addreplacerg | adds or replaces RG tags |
| 2015-06-11 | 1.3 | quickcheck | quickly check if SAM/BAM/CRAM file appears intact |
| 2015-08-24 | 1.3 | fasta | converts a BAM to a FASTA |
| 2017-08-08 | 1.6 | markdup | mark duplicates |
| 2018-05-21 | 1.9 | fqidx | index/extract FASTQ |
| 2018-12-11 | 1.1 | coverage | alignment depth and percent coverage |
| 2020-04-09 | 1.11 | ampliconclip | clip oligos from the end of reads |
| 2020-04-30 | 1.11 | ampliconstats | generate amplicon specific stats |

**Table S2.** **Table of BCFtools commands**

List of BCFtools commands and plugins (prefixed with '+') with the date and version of the initial commit.

Marked are contributions by David Laehnemann (*), Nicola Asuni (+) and Giulio Genovese (#).

| **Date** | **Version** | **Command** | **Description** |
| --- | --- | --- | --- |
| 2012-05-17 | 0.1.0 | view | VCF/BCF conversion, view, subset and filtering |
| 2012-08-07 | 0.1.0 | merge | merge VCF/BCF files files from non-overlapping sample sets |
| 2012-09-06 | 0.1.0 | isec | intersections of VCF/BCF files |
| 2013-02-07 | 0.1.0 | query | transform VCF/BCF into user-defined formats |
| 2013-02-12 | 0.1.0 | filter | filter VCF/BCF files using fixed thresholds |
| 2013-03-04 | 0.1.0 | gtcheck | check sample concordance, detect sample swaps and contamination |
| 2013-03-12 | 0.1.0 | norm | left-align and normalize indels, and more |
| 2013-08-20 | 0.1.0 | call | -m and -c calling |
| 2013-08-30 | 0.1.0 | stats | produce VCF/BCF stats |
| 2013-11-05 | 0.2.0 | annotate | annotate and edit VCF/BCF files |
| 2013-11-05 | 0.2.0 | roh | identify runs of autozygosity (HMM) |
| 2014-01-10 | 0.2.0 | +missing2ref | sets missing genotypes ("./.") to ref allele ("0/0" or "0\|0") |
| 2014-01-14 | 0.2.0 | concat | concatenate VCF/BCF files from the same set of samples |
| 2014-02-07 | 0.2.0 | index | index VCF/BCF files |
| 2014-04-11 | 0.2.0 | +counts | minimal plugin which counts number of SNPs, Indels, and total number of sites |
| 2014-04-11 | 0.2.0 | +dosage | prints genotype dosage |
| 2014-04-23 | 0.2.0 | +frameshifts | annotate frameshift indels |
| 2014-07-01 | 0.2.0 | reheader | modify VCF/BCF header, change sample or chromosome names |
| 2014-07-29 | 0.2.0 | convert | convert VCF/BCF files to different formats and back |
| 2014-09-02 | 1.0 | cnv | CNV calling from array data (HMM) |
| 2014-09-03 | 1.0 | polysomy | detect number of chromosomal copies from Illumina's B-allele frequency |
| 2014-09-25 | 1.1 | +fixploidy | sets correct ploidy |
| 2014-10-01 | 1.1 | consensus | create consensus sequence by applying VCF variants |
| 2015-01-08 | 1.1 | +tag2tag | convert between similar tags, such as GL and GP |
| 2015-07-28 | 1.2 | +fill-tags | set various INFO tags |
| 2015-09-16 | 1.2 | +setGT | set genotypes according to rules requested by the user |
| 2015-10-02 | 1.2 | +color-chrs | color shared chromosomal segments, requires trio VCF with phased GTs |
| 2015-10-02 | 1.2 | +impute-info | add imputation information metrics to the INFO field based on selected FORMAT tags |
| 2015-10-02 | 1.2 | +mendelian | count Mendelian consistent / inconsistent genotypes |
| 2016-02-23 | 1.3 | +GTisec (*) | count genotype intersections across all possible sample subsets in a vcf file |
| 2016-06-21 | 1.3.1 | +guess-ploidy | determine sample sex by checking genotype likelihoods (GL,PL) or genotypes (GT) |
| 2016-06-28 | 1.3.1 | +ad-bias | find positions with wildly varying ALT allele frequency (Fisher test on FMT/AD) |
| 2016-06-28 | 1.3.1 | +trio-switch-rate | calculate phase switch rate in trio samples, children samples must have phased GTs |
| 2016-08-02 | 1.3.1 | +af-dist | collect AF deviation stats and GT probability distribution given AF and assuming HWE |
| 2016-08-03 | 1.3.1 | +fixref | determine and fix strand orientation |
| 2016-08-03 | 1.3.1 | +GTsubset (*) | output only sites where the requested samples all exclusively share a genotype |
| 2016-08-05 | 1.3.1 | mpileup | moved from samtools |
| 2016-09-02 | 1.3.1 | csq | call variation consequences |
| 2016-09-21 | 1.3.1 | +fill-from-fasta | fill INFO or REF field based on values in a fasta file |
| 2016-11-23 | 1.3.1 | +isecGT | compare two files and set non-identical genotypes to missing |
| 2017-04-21 | 1.4 | +check-sparsity | print samples without genotypes in a region or chromosome |
| 2017-05-17 | 1.4.1 | +prune | prune sites by missingness, allele frequency or linkage disequilibrium |
| 2017-06-29 | 1.5 | +check-ploidy | check if ploidy of samples is consistent for all sites |
| 2017-07-06 | 1.5 | sort | sort VCF/BCF file |
| 2017-11-12 | 1.6 | +split | split VCF by sample, creating single- or multi-sample VCFs |
| 2018-03-15 | 1.7 | +contrast | simple association test, checks for novel alleles and genotypes in two groups of samples |
| 2018-05-18 | 1.8 | +trio-stats | calculate transmission rate in trio children |
| 2018-06-28 | 1.8 | +smpl-stats | calculates basic per-sample stats |
| 2018-07-04 | 1.8 | +add-variantkey (+) | add VariantKey INFO fields VKX and RSX |
| 2018-07-04 | 1.8 | +allele-length (+) | count the frequency of the length of REF, ALT and REF+ALT |
| 2018-07-04 | 1.8 | +variantkey-hex (+) | generate unsorted VariantKey-RSid index files in hexadecimal format |
| 2018-10-04 | 1.9 | +gvcfz | compress gVCF file by resizing non-variant blocks according to specified criteria |
| 2019-03-06 | 1.9 | +remove-overlaps | remove overlapping variants and duplicate sites |
| 2019-03-25 | 1.9 | +split-vep | extract fields from structured annotations such as INFO/CSQ created by bcftools/csq or VEP |
| 2019-04-24 | 1.9 | +parental-origin | determine parental origin of a CNV region |
| 2019-04-29 | 1.9 | +indel-stats | calculates per-sample or de novo indels stats |
| 2020-06-23 | 1.10.2 | +scatter (#) | intended as an inverse to `bcftools concat`, scatter VCF by chunks or regions, creating multiple VCFs |
| 2020-12-16 | 1.11 | +trio-dnm2 | screen variants for possible de-novo mutations in trios |
