# Supplementary material for: Twelve years of SAMtools and BCFtools
Source: Gigascience. 2021 Feb 16;10(2):giab008. doi: 10.1093/gigascience/giab008 (PMC7931819; doi:10.1093/gigascience/giab008)
Supplement: giab008_Response_to_Reviewer_Comments_Original_Submission [file giab008_response_to_reviewer_comments_original_submission.pdf]

Author's Response To Reviewer Comments

Close

Submission ID: GIGA-D-20-00369
Title: Twelve years of SAMtools and BCFtools

Response to Reviewers

We would like to thank the editor and reviewers for careful review of our manuscript. Their suggestions and
comments were helpful for us to refine this paper. Please see the list of revisions and our responses highlighted below.

Sincerely,
Andrew Whitwham et al.

Reviewer #1: Danecek et al. summarized the status, new features, and improvements in the SAMtools and BCFtools. They described the details in performance optimization, the new design of the separation of IO and CPU intensive tasks, and the separation of HTSlib from the tools. They also discussed the benefit of adopting software engineering techniques into SAMtools and BCFtools development. The paper is well written in clear language. The software is widely used and well documented.

I only have a few minor observations regarding the manuscript.
1. It would be clearer to rephrase or explain the problem these tools addressed in the Abstract section. Although the tools themselves speak a lot, it would still be friendly to a broader audience.

Thank you for the suggestion. We updated the text to describe that the programs include tools for file format conversion and manipulation, sorting, querying, statistics, variant calling and effect analysis amongst other methods.

2. The transition of the first paragraph of the BCFtools section is not smooth. Consider moving the second sentence to the next paragraph.

We have restructured according to your suggestion, thank you.

3. As the authors pointed out, there might be suboptimal usages among third-party tutorials or home-brewed pipelines. It would be helpful to provide flowcharts with "best practices" for typical scenarios.

Thank you, that's a good point. We have added links to the workflows and documentation:

"We encourage readers to follow best practices and workflows published at
https://www.htslib.org/workflow/"

and

"Full documentation for these commands is included with the package in the form of UNIX man pages, and can also be found online together with short tutorials, math notes, and other documentation at
https://samtools.github.io/bcftools"

Reviewer #2: Just a few very minor nits to pick:

Background paragraph 3: "the release 0.1.4" -> "release 0.1.4"

Fixed, thank you.

Findings paragraph 2: A table with ~1 sentence summaries of what these commands currently do would be nice here. (Given that this is a "12 years" retrospective article, this table could also include the date or version number where each command was introduced.)

Thank you for the suggestion, it is a good idea. We added supplementary tables with SAMtools and BCFtools commands and plugins.

BCFtools paragraph 6: Can add a reference regarding how variant caller accuracy and speed compares to today's other most commonly used tools. Could do the same for the ROH caller.

Although the intention for this article is to describe recent updates rather than act as a comparison to other tools, we have added references to a few comparison articles. We'd be happy to include more references if we missed them in literature.

Discussion paragraph 3: "UndefinedBehaviourSanitizer" -> "UndefinedBehaviorSanitizer"

Fixed, thank you.

Discussion paragraph 4: First sentence should be rewritten to have correct grammar.

This sentence has now been revised. Thank you.

Discussion paragraph 5: "species, vertebrate, non-vertebrates," -> "species: vertebrates, non-vertebrates,"
"future developments" -> "future development"

Fixed, thank you.

Close
