# Supplementary material for: Twelve years of SAMtools and BCFtools
Source: Gigascience. 2021 Feb 16;10(2):giab008. doi: 10.1093/gigascience/giab008 (PMC7931819; doi:10.1093/gigascience/giab008)
Supplement: giab008_Reviewer_1_Report_Original_Submission — Fan Zhang -- 1/3/2021 Reviewed [file giab008_reviewer_1_report_original_submission.pdf]

Reviewer Report

Title: Twelve years of SAMtools and BCFtools

Version: Original Submission **Date:** 1/3/2021

Reviewer name: Fan Zhang

Reviewer Comments to Author:

Danecek et al. summarized the status, new features, and improvements in the SAMtools and BCFtools. They described the details in performance optimization, the new design of the separation of IO and CPU intensive tasks, and the separation of HTSlib from the tools. They also discussed the benefit of adopting software engineering techniques into SAMtools and BCFtools development. The paper is well written in clear language. The software is widely used and well documented.
I only have a few minor observations regarding the manuscript.
1. It would be clearer to rephrase or explain the problem these tools addressed in the Abstract section. Although the tools themselves speak a lot, it would still be friendly to a broader audience.
2. The transition of the first paragraph of the BCFtools section is not smooth. Consider moving the second sentence to the next paragraph.
3. As the authors pointed out, there might be suboptimal usages among third-party tutorials or home-brewed pipelines. It would be helpful to provide flowcharts with "best practices" for typical scenarios.

Level of Interest

Please indicate how interesting you found the manuscript: Choose an item.

Quality of Written English

Please indicate the quality of language in the manuscript: Choose an item.

Declaration of Competing Interests

Please complete a declaration of competing interests, considering the following questions:

- Have you in the past five years received reimbursements, fees, funding, or salary from an organisation that may in any way gain or lose financially from the publication of this manuscript, either now or in the future?
- Do you hold any stocks or shares in an organisation that may in any way gain or lose financially from the publication of this manuscript, either now or in the future?
- Do you hold or are you currently applying for any patents relating to the content of the manuscript?

- Have you received reimbursements, fees, funding, or salary from an organization that holds or has applied for patents relating to the content of the manuscript?
- Do you have any other financial competing interests?
- Do you have any non-financial competing interests in relation to this paper?

If you can answer no to all of the above, write 'I declare that I have no competing interests' below. If your reply is yes to any, please give details below.

I declare that I have no competing interests

I agree to the open peer review policy of the journal. I understand that my name will be included on my report to the authors and, if the manuscript is accepted for publication, my named report including any attachments I upload will be posted on the website along with the authors' responses. I agree for my report to be made available under an Open Access Creative Commons CC-BY license (http://creativecommons.org/licenses/by/4.0/). I understand that any comments which I do not wish to be included in my named report can be included as confidential comments to the editors, which will not be published.

Choose an item.

To further support our reviewers, we have joined with Publons, where you can gain additional credit to further highlight your hard work (see: https://publons.com/journal/530/gigascience). On publication of this paper, your review will be automatically added to Publons, you can then choose whether or not to claim your Publons credit. I understand this statement.

Yes Choose an item.
